# Nanoencapsulation Enhances Anticoagulant Activity of Adenosine and Dipeptide IleTrp

**DOI:** 10.3390/nano9091191

**Published:** 2019-08-23

**Authors:** Trung Dinh Nguyen, The Ngoc Nguyen, Trang Thuy Thi Nguyen, Igor A. Ivanov, Khoa Cuu Nguyen, Quyen Ngoc Tran, Anh Ngoc Hoang, Yuri N. Utkin

**Affiliations:** 1 Institute of Research and Development, Duy Tan University, Da Nang City 550000, Vietnam; 2 Institute of Applied Materials Science, Vietnam Academy of Science and Technology, Ho Chi Minh City 700000, Vietnam; 3 Graduate University of Science and Technology, Vietnam Academy of Science and Technology Ho Chi Minh City 700000, Vietnam; 4 Tra Vinh University, Tra Vinh City 940000, Vietnam; 5 Faculty of Pharmacy, Nguyen Tat Thanh University, Ho Chi Minh City 700000, Vietnam; 6 Shemyakin-Ovchinnikov Institute of Bioorganic Chemistry, Russian Academy of Science, 119991 Moscow, Russia

**Keywords:** anticoagulant, adenosine, heparin, pluronic P123, nanogel

## Abstract

It is well-known that drugs administered into an organism intravenously or through the gastrointestinal tract are degraded by enzymes of the body, reducing their therapeutic effect. One of the ways to decrease this undesirable process is through the inclusion of drugs in nanomaterials. Earlier strong anticoagulant activity was demonstrated for dipeptide IleTrp (IW) and adenosine (Ado). In this work, the effect of inclusion in nanomaterials on the biological activity of IW and Ado was studied. For this purpose, Ado and IW were incorporated into thermosensitive nanogel composed of pluronic P123-grafted heparin. The prepared nanocarrier was characterized by transmission electron microscopy, dynamic light scattering, and ζ-potential. Biological activity was determined by measuring the bleeding time from mouse tail in vivo and the time of clot formation in vitro. It was found that encapsulation of Ado and IW into nanomaterial significantly increased their effects, resulting in an increase in the bleeding time from mouse tail and clot formation time. Thus, inclusion of low molecular weight anticoagulants Ado and IW into nanomaterials may be considered a way to increase their biological activity.

## 1. Introduction

One of the main problems with the use of drugs is their degradation in the body, requiring an increase in the drug concentration to achieve a therapeutic effect; however, increasing the concentration can lead to a number of side effects [1,2,3]. Increasing stability is particularly important for peptide drugs. Over the last decade, peptides have been more widely used in medicine and biotechnology, and research on peptides with therapeutic potential are developing intensively. The advantages of peptides are good efficacy, safety and tolerability, high selectivity, and predictable metabolism. However, there are a number of shortcomings that limit their use. Peptides are less stable than ordinary organic compounds, they are prone to hydrolysis and oxidation, have a short lifetime in the body, and are quickly eliminated [4,5]. One of the ways to overcome these disadvantages is the use of peptides in combination with nanomaterials [6,7]. Nanomaterials allow for modifying the properties of compounds without reducing their biological activity. Thus, polymer-based nanoparticles enable physical protection from environmental stimuli and may target the load to specific sites [8]. Making these systems stimuli-responsive is an efficient way to improve their functions. Use of stimulus-sensitive polymers for the manufacture of smart drug delivery systems and/or active targeting delivery systems may greatly increase the therapeutic efficacy of drugs and biologically active molecules [9,10].

Among many other nanomaterials, nanogels can be used as biodegradable and highly efficient carriers for the transportation of drugs and controlled drug release [11,12]. Encapsulation in nanogels is widely used to enhance solubility and stability, e.g., for anticancer drugs. This is especially important for the peptides; loading peptides into nanogel increases their stability and reduces acute toxicity. In fact, several types of nanocarriers have performed their roles in the field very well. Thus, poly(lactide-co-glycolide)-poly(ethylene imine) nanoparticles effectively delivered superoxide dismutase to the cytoplasm via direct translocation and endocytosis-endosomal escape pathways [6]. Mesoporous silica-based nanoparticles have been used for encapsulation and targeted delivery of several proteins and peptides [13]. Along this line, heparin and its derivatives were utilized to deliver many kinds of drugs or bioactive molecules via heparin-drug conjugate, drug-loaded polymeric nanoparticles or nanogels, nanosized complexation, and heparin-coated organic or inorganic nanoparticles [14,15,16]. These heparin-based carriers have been used in various applications, thereby improving the bioavailability and the therapeutic efficacy of drugs or biologically active compounds. The anticoagulant properties of the heparin itself were improved by covalent attachment to the outer surface of the colloidal mesoporous silica nanoparticles [17]. The nanosystem obtained may be used for incorporation of biologically active molecules as deliverable cargo. Recently designed heparin-pluronic nanogels have been applied as sustained-release delivery systems for several drugs. They were successfully used for encapsulation and delivery of such proteins as acidic fibroblast growth factor [18] and nerve growth factor [19]. Heparin-pluronic nanogels were reported to demonstrate the ability to stabilize and control the release of even comparatively large proteins such as monoclonal antibodies (3D8 scFv) [20].

Blood coagulation disorders are life threatening conditions which require medical intervention. At present, many highly effective anticoagulants are on the market [21]; however, there are some problems to be solved. For example, application of anticoagulants that are stable in the organism require treatment with reversal agents, while the degradable drugs are not so effective. In this respect, nanomaterials may help greatly by stabilizing otherwise unstable drugs; in addition, they can provide sustained drug release. Thus, a low molecular weight anticoagulant enoxaparin was encapsulated in biodegradable nanoparticles of poly (ε-caprolactone) [22]. Sustained release of the drug from polymeric nanoparticles was observed for a greater period than after injection of enoxaparin. These results indicate prospects for the use of nanomaterials in clinical practice.

Earlier in the venom of *Heterometrus laoticus* scorpion, the dipeptide IleTrp (IW) and adenosine (Ado), both exhibiting anticoagulant properties were found [23]. In this paper, it is reported that the encapsulation of Ado and IW in the pluronic P123-grafted heparin (Hep-P123) nanomaterials significantly enhanced their anticoagulant properties, providing sustained drug release over time. It should be noted that Ado plays an important role in the cardiovascular system [24]. It is used as a drug for treating several heart disorders; in particular, irregular heart rhythm. However, Ado has an extremely short half-life in vivo, and so therefore its encapsulation in Hep-P123 may improve drug application.

## 2. Materials and Methods

### 2.1. Chemicals

Adenosine (Ado) was from Merck KGaA (Darmstadt, Germany). Dipeptide IW was prepared as in [23]. Dialysis membranes (molecular weight cut-off of 14 and 3.5 kDa) were supplied by Repligen Corporation (Rancho Dominguez, CA, USA).

### 2.2. Mice

Swiss albino male mice were obtained from the Nha Trang Institute of Vaccines and Medical Biological Preparations. The mice were kept for at least 2 days prior to the test at Faculty of Pharmacy, Nguyen Tat Thanh University. All of the appropriate actions were taken to minimize discomfort in the mice. World Health Organization’s International Guiding Principles for Biomedical Research Involving Animals were followed during experiments on animals.

Animal experiments described in this study were approved by the Scientific Council of the Faculty of Pharmacy, Nguyen Tat Thanh University (Protocol No 1). Protocol was signed by the Chairman of the Council, Vice Principal Prof. Nguyen Van Thanh and the Council Secretary Dr. Vo Thi Ngoc My. Date of approval was 19 January, 2017.

### 2.3. Preparation of Pluronic P123-grafted Heparin (Hep-P123)

Hep-P123 was prepared at the Institute for Applied Materials Science by described procedure [25]. Hep-P123 copolymer was synthesized via conjugation of the pluronic P123 partially activated with *p*-nitrophenyl chloroformate with the heparin aminated with 1,4-diaminobutane. The synthesized Hep-P123 copolymer was purified by one-week dialysis using a cellulose membrane (molecular weight cut-off of 14 kDa) against water and then lyophilized to obtain dry nanomaterial. The copolymer structure was confirmed by proton nuclear magnetic resonance (^1^H-NMR) using Bruker AM500 FT NMR spectrometer (Bruker, Billerica, MA, USA) and thermal gravimetric analysis (TGA) using Thermogravimetric analyzer (Mettler-Toledo, Columbus, OH, USA). TGA was performed with a heating rate of 10 °C/min from room temperature to 700 °C. For the material protection, argon gas at a flow rate of 40 mL/min was used during sample analysis by TGA.

### 2.4. Preparation of IW and Ado loaded Hep-P123 Nanogels

Hep-P123 loaded with IW and Ado was prepared as described [26]. In brief, to encapsulate IW and Ado, Hep-P123 copolymer (25 mg) was dissolved completely in 2.5 mL water at 15–20 °C. Then, an aqueous IW (1.38 mg in 0.08 mL) or Ado (2.5 mg in 0.04 mL) solution was added drop-wise. The solution mixture was stirred overnight (250 rpm), and finally the reaction mixtures were dialyzed against distilled water and then freeze-dried to obtain desiccated forms of Hep-P123-IW and Hep-P123-Ado.

The amounts of IW and Ado encapsulated in nanomaterials were determined by UV spectrophotometry using an Agilent 8453 spectrophotometer (Agilent Technologies, Santa Clara, CA, USA). IW and Ado loaded Hep-P123 were dialyzed against water, the optical density of dialysate was measured at 280 nm for IW and 260 nm for Ado, and the amount of substances released from the dialysis bag was calculated. Basing on these data, the encapsulated amounts of IW and Ado in the nanocarriers were indirectly determined. The drug loading (DL%) in Hep-P123 and entrapment efficiency (EE%) were calculated using Equations (1) and (2) [27]:

(1) $$\%EE=\frac{W_{total\;\mathrm{drug}}-W_{free\;\mathrm{drug}}}{W_{total\;\mathrm{drug}}}*100\%$$

(2) $$\%DL=\frac{W_{total\;\mathrm{drug}}-W_{free\;\mathrm{drug}}}{W_{Hep-P123}}*100\%$$

where *W_total drug_* is the total weight of IW or Ado, *W_free drug_*—weight of free IW or Ado, and *W_Hep-P123_*—weight of the grafted copolymer.

The surface charge and particle size of the complexed nanoparticles were analyzed by HORIBA SZ-100 nanoparticle size analyzer (Horiba Ltd., Kyoto, Japan). The scattered light was received at two angles, 90° or 173°. Stokes-Einstein equation was used to calculate particle size. The size and shape of nanoparticles were studied by transmission electron microscopy using JEM-1400 instrument (JEOL Ltd., Tokyo, Japan) as described [25]. For sample preparation, 1 µL solution was dropped on a net frame and dried at room temperature. After drying, the frame was dipped into uranyl acetate 1% w/v solution and shaken gently. Samples were measured at 25 °C.

### 2.5. Study of IW and Ado Release Behavior

To study the release of anticoagulant from Hep-P123, a modified dialysis method was used. Aqueous Hep-P123-Ado or Hep-P123-IW solution (1 mL) was put in cellulose dialysis tubing (MWCO 3.5 kDa) which was immersed in PBS buffer (10 mL, pH 7.4, 37 °C) under stirring at 100 rpm. At predetermined time intervals, 1 mL of buffer was removed and replaced with the same volume of pre-warmed PBS medium. The experiment was repeated in triplicate. Anticoagulant release was quantified by measuring optical density at 280 nm for IW and 260 nm for Ado using Agilent 8453 Spectrophotometer. The cumulative anticoagulant release was calculated using the following formula:

(3) $$Q=C_{n}V_{s}+V_{t}\sum_{i=1}^{n-1}C_{n-1}$$

where C_n_ is the concentration of drug in the sample, C_n-1_ is the concentration of drug released at time *t*, V_s_ is the volume of incubation medium, and V_t_ is the volume of medium replaced at time t [27,28]. 

### 2.6. Determination of Anticoagulant Activity

Determination of anticoagulant activity was done as described [23]. In brief, the solution containing encapsulated anticoagulant in 0.9% NaCl was injected in lateral veins of mouse tails at a dose of 2.48 mg/kg calculated for IW or Ado (injection volume 0.1 mL per 10 g of mouse body weight). The blood coagulation time was determined by modified Burker method [29]. In brief, a drop of blood from the mouse tail was placed on the glass. Every 30 seconds one tried to tear it away from the glass with the help of an injection needle. The moment when the formed fibrin threads could detach the blood clot from the glass corresponded to the end of the coagulation. Tail bleeding times were measured using the method described by Liu et al. [30]. The distal 5 mm of tail was amputated and the tail (diameter of about 1.5 mm) was immersed in 37 °C solution of 0.9% NaCl. Time to visible cessation of bleeding was recorded.

### 2.7. Statistical Analysis

Significance in differences between experimental and control groups was analyzed by Kruskal-Wallis method and then by Mann-Whitney method using Minitab 15.0 (Minitab, LLC, State College, PA, USA)) program. The differences were considered significant for *P* values < 0.05. All results are presented as the mean ± SEM (standard error of the mean). Charts were drawn with the program SigmaPlot 12.0.

## 3. Results

### 3.1. Characterization of Hep-P123 Copolymer

**Figure 1:**
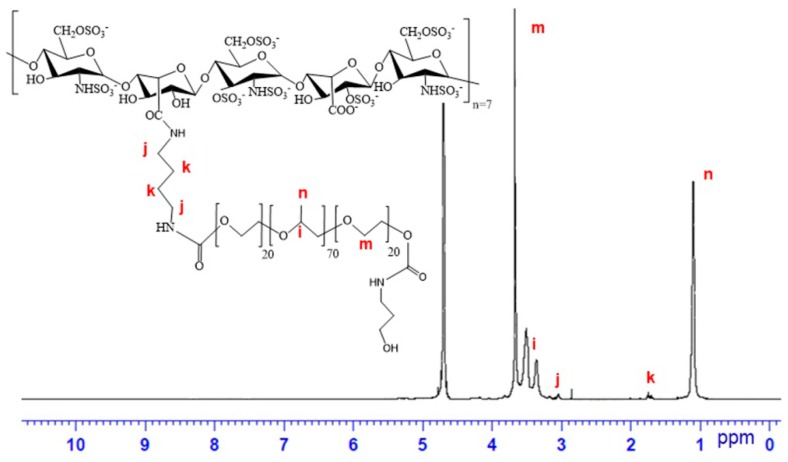
^1^H-NMR spectrum of Hep-P123 copolymer. Letters on the spectrum indicate to which protons in the Hep-P123 molecule the signals correspond.

The Hep-P123 copolymer used in this study was characterized by ^1^H-NMR spectroscopy and TGA. The ^1^H-NMR spectrum is shown in Figure 1. The signals of methylene protons at *δ* = 1.74 ppm (k) and *δ* = 3.03 ppm (j) assigned to diaminobutyl moieties connecting P123 to heparin are observed. Besides, the intensive signals of proton of methyl group (at *δ* = 1.105 ppm, n) in polypropylene oxide (PPO) and methylene group (at *δ* = 3.676 ppm, m) in polyethylene oxide (PEO) are present. As the number of protons in heparin is much smaller than the number of protons in grafted P123, their signals are also significantly weaker. They are observed at *δ* = 1.98 and 3.22 ppm. So, ^1^H-NMR spectrum demonstrates the presence of both P123 and heparin in Hep-P123.

**Figure 2:**
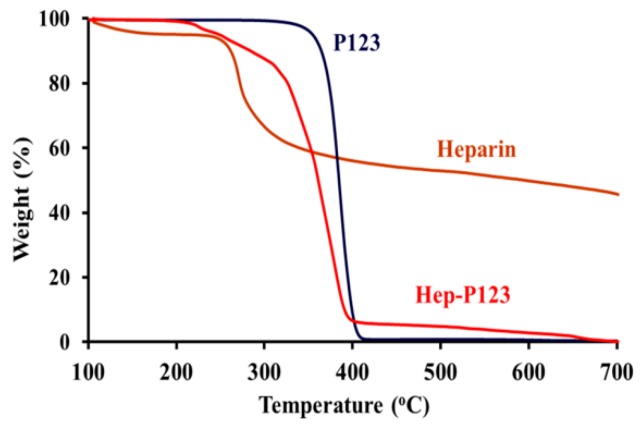
Thermal gravimetric analysis of P123, heparin and Hep-P123 copolymer.

The results of TGA (Figure 2) show that up to 220 °C both pluronic P123 and heparin were stable without substantial adsorbed water loss, which usually occurs at a temperature above 100 °C. However, the decomposition of heparin progressed sharply at 260 °C and then more slowly from 400 °C to 700 °C, while the total mass of pluronic P123 was lost completely above 420 °C. Hep-P123 started to lose mass at above 200 °C with a sharp mass loss between 300 °C and 400 °C. However, the decomposition was not complete and similarly to heparin continued slowly up to 700 °C. This behavior indicated the presence of heparin in copolymer. Based on the data obtained, the content of heparin grafting P123 in Hep-P123 was calculated as 27.25 wt/wt%., using the method described earlier [31,32].

### 3.2. Preparation of IW and Ado Anticoagulants Encapsulated in Hep-P123 Nanogels

**Figure 3:**
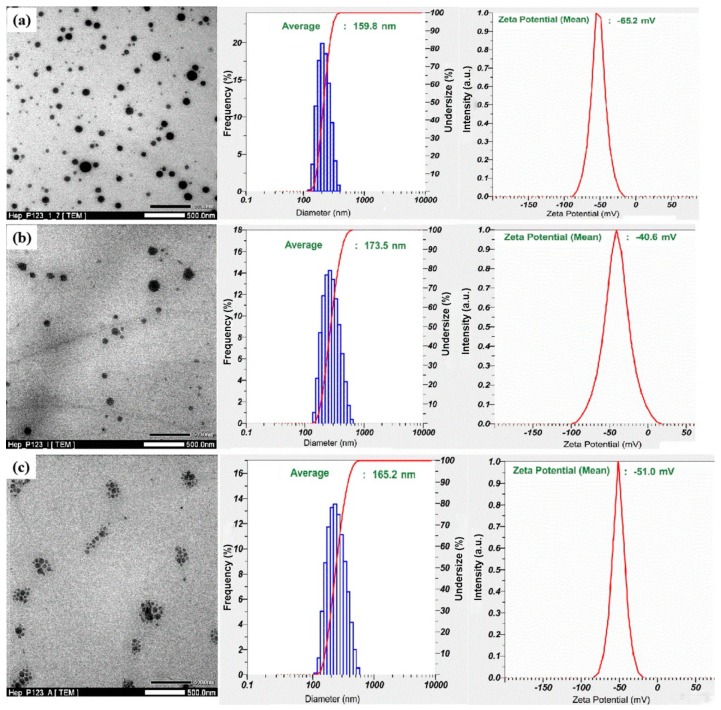
Transmission electron microscopy (TEM) images (left panels), dynamic light scattering (DLS) data (middle panels) and Zeta potential (right panel) of Hep-P123 (**a**) Hep-P123-IW (**b**) and Hep-P123-Ado (**c**).

**Figure 4:**
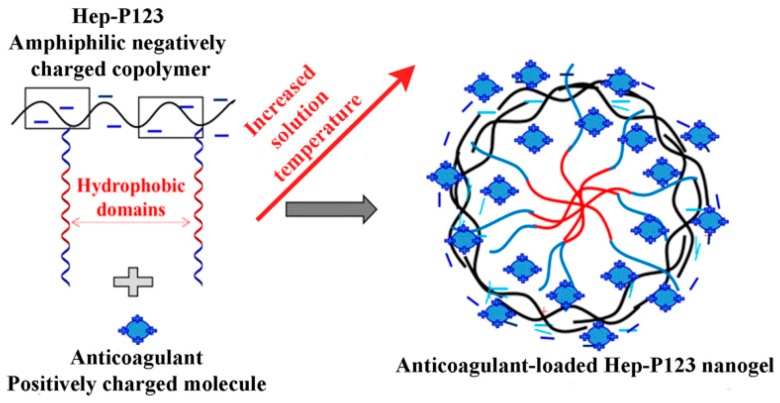
Illustrated mechanism for formation of Ado or IW-loaded Hep-P123 nanogels.

The IW and Ado anticoagulants were encapsulated in the Hep-P123 nanocarrier as described [26]. The nanoparticles obtained were characterized by transmission electron microscopy (TEM), dynamic light scattering (DLS) and zeta potential measurements (Zeta) (Figure 3). TEM data show that size distribution of the nanoparticles ranges from 20 to 150 nm by. DLS results indicate that encapsulation of both IW and Ado led to a slight increase in particle size distribution of the Hep-P123 as compared to its unoccupied form. Zeta potential of the drug-loaded platform significantly increased from −65.2 mV for Hep-P123 nanogel to −40.6 mV and −51.0 mV for IW- and Ado-loaded Hep-P123, respectively. This increase may be explained by the presence of free amino groups in Ado and IW that under interaction with negatively charged Hep-P123 could be protonated, resulting in a decrease of total negative charge of the loaded nanocarrier molecule (Figure 4). Electrostatic interaction may enhance drug entrapment efficiency (EE), which is equal to 95.66 and 69.76 wt% for IW and Ado, respectively.

### 3.3. Release Profiles of Ado and IW

**Figure 5:**
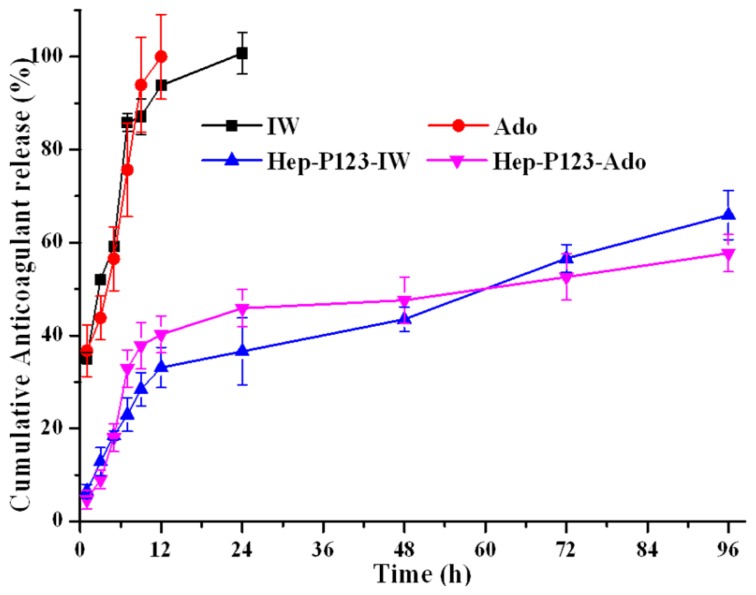
Release profiles of the free and encapsulated in Hep-P123 anticoagulants at physiological conditions pH 7.4 (37 ± 1 °C; *n*=3).

Drug release studies in vitro are important to help with an understanding of the bioavailability of encapsulated agents through intravenous administration. We have studied the in vitro release of Ado, IW, Hep-P123-Ado and Hep-P123-IW through a dialysis membrane over the course of 96 hours, the results of which are shown in Figure 5. A fast release of intact Ado and IW from a dialysis bag was observed and 43.8% of Ado and 52.1% of IW were discharged over 3 h (Table 1). These values kept increasing, rapidly approaching 76% and 86% after 7 h of observation. After 24 h, both Ado and IW were released completely. 

However, in the case of the anticoagulants encapsulated in Hep-P123, the release values after 7 h were only 32.9% and 23.0% for Hep-P123-Ado and Hep-P123-IW, respectively; after 24 hours these values were equal to 45.9% and 36.6%, and even after 96 hours the release was not complete, reaching 57.7% and 66.0% for Hep-P123-Ado and Hep-P123-IW, respectively. As can be seen in Figure 5, the release process is biphasic. The initial rapid release rate may typically be associated with a detachment of the compounds from the surface of Hep-P123, while the slow one is the result of a sustained drug release from the inside of the nanogel. The in vitro release of the bioactive compounds was mainly driven by a diffusion-controlled mechanism. The results of these experiments show good potential of the Hep-P123 nanogel in controlled anticoagulant release.

### 3.4. Studies of Anticoagulant Activity

#### 3.4.1. Influence on Blood Coagulation in vitro

**Figure 6:**
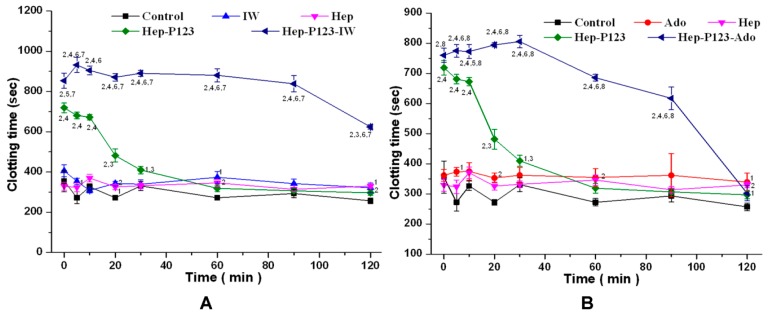
Whole blood coagulation time. Influence of the IW (**A**) and Ado (**B**) as well as their encapsulated forms on the clotting time in comparison with Hep and Hep-P123. The abscissa indicates the time after injection. M ± m. *n* = 9 for each group. The numbers near the points indicate the probability values (*p*). 1 − *p* < 0.05 and 2 − *p* < 0.01 compared with control. 3 − *p* < 0.05 and 4 − *p* < 0.01 compared with Hep. 5 − *p* < 0.05 and 6 − *p* < 0.01 compared with Hep-P123. 7 − *p* < 0.01 compared with IW. 8 − *p* < 0.01 compared with Ado.

To study the blood coagulation in vitro after drug injection, a drop of blood was taken from the mouse tail vein and the coagulation time was determined using the Burker method [29]. To study anticoagulant activity, the encapsulated anticoagulants were injected into the lateral vein of the mouse tail at a dose of 2.48 mg/kg of IW or Ado calculated based on drug loading in Hep-P123. The effects of Ado, IW, Heparin, Hep-P123, Hep-P123-IW, and Hep-P123-Ado were studied. The clotting time was determined within two hours after injection. Figure 6 shows that the injection of Ado and IW resulted in increased clotting time. Injection of Hep led to an increase of clotting time as well. At all tested times, Hep-P123 demonstrated the clotting time longer than in the control group. However, after 30 minutes of observation, this difference had no statistical significance. In comparison to Heparin, Hep-P123 caused a longer blood clotting time up to the thirtieth minute, while after this time no difference with Hep was found. However, the largest increase in the clotting time was observed when the anticoagulants were encapsulated in Hep-P123. Thus, at all observation times up to minute 120, Hep-P123-IW caused a clotting time longer than in the control group as well as in IW, Hep, and Hep-P123 treated groups. The Ado effect on blood clotting was greatly enhanced by encapsulation in Hep-P123. However, the action of Hep-P123-Ado was not as long as that of Hep-P123-IW. Its effect was not distinguished from control after 120 minutes. In general, Hep-P123-IW and Hep-P123-Ado caused a longer blood clotting time than the Ado, IW, Hep and Hep-P123. It can be concluded that IW and Ado encapsulation in Hep-P123 nanogels strongly increase the clotting time and prolong the anticoagulant effect of dipeptide.

#### 3.4.2. Influence on Bleeding Time in vivo

**Figure 7:**
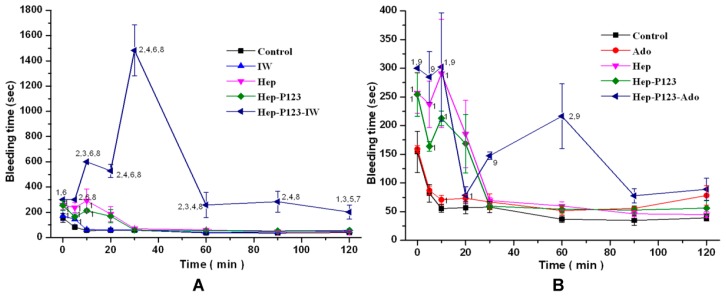
Bleeding time. Influence of the IW (**A**) and Ado (**B**) as well as their encapsulated forms on the bleeding time in comparison with Hep and Hep-P123. M ± m. *n* = 9 for each group. The numbers near the points indicate the probability values (*p*). 1 − *p* < 0.05 and 2 − *p* < 0.01 compared with control. 3 − *p* < 0.05 and 4 − *p* < 0.01 compared with Hep. 5 − *p* < 0.05 and 6 − *p* < 0.01 compared with IW. 7 − *p* < 0.05 and 8 − *p* < 0.01 compared with Hep-P123. 9 − *p* < 0.01 compared with Ado.

To determine in vivo anticoagulant activity, free and encapsulated anticoagulants were injected in the mice as described above and tail bleeding times were evaluated. The bleeding time was determined within two hours after injection and the data obtained are shown in Figure 7. As it was shown earlier [23], at all times tested, the injection of Ado and IW resulted in substantial increase in the bleeding time. Injection of heparin led to an increase in bleeding time only for the first 10 min. Hep-P123 demonstrated the bleeding time longer than in the control group only for the first 10 min as well. No difference was observed in comparison to heparin. The largest increase in the bleeding time was observed when the anticoagulants Ado and IW were encapsulated in Hep-P123. Thus, at all observation times up to minute 120, Hep-P123-IW caused a bleeding time longer than in the control group as well as in IW, Hep, and Hep-P123 treated groups (Figure 7A). It can be seen that IW encapsulation strongly increased the bleeding time and prolonged the anticoagulant effect of dipeptide. In particular, prolonged bleeding was registered during the first 30 minutes of the observation, especially at the 30th minute. At this point, the bleeding time was about 22-times longer compared with Hep and about 26-times longer compared with the control group. In comparison to Hep-P123, the Hep-P123-IW caused a longer bleeding time at all times of the test and these differences were statistically significant.

The effect of encapsulation on Ado activity was not as pronounced as in the case of IW. However, at all tested times, Hep-P123-Ado was shown to cause a bleeding time longer than in the control group, but only at the 10th and 30th minutes, the bleeding time was significantly longer and this difference was of statistical significance (Figure 7B). For the rest of the time, these differences had no statistical significance. Compared to Ado, Hep-P123-Ado causes a longer bleeding with statistically significant difference up to the 60th minute. Compared to Hep and Hep-P123, Hep-P123-Ado caused a longer bleeding at some observation times, but these differences were not statistically significant (Figure 7B).

## 4. Discussion

Nanomaterials possessing unique properties have good prospects for medical applications. In particular, they can be used as carriers for the delivery of poorly soluble drugs or for stabilization of easily degradable medicines. In this work, we used nanomaterials for the protection of easily degradable anticoagulants to prolong their effects.

In this work, for encapsulation of anticoagulants, we used the nanogel composed of pluronic P123 and heparin. It should be noted that when entering the bloodstream nanomaterials themselves can affect hemostasis [33] and the effects produced depend on nanomaterial nature. Heparin is a well-known anticoagulant and its anticoagulating mechanism is based primarily on binding to antithrombin III, which serves as a physiological inhibitor of activated blood coagulation factors IXa, Xa, XIa, XIIa, and thrombin [34]. The heparin binding to antithrombin III enhances its inhibitory activities against coagulation factors, most importantly factors IXa, Xa, and thrombin, which are serine proteases resulting in an increase of blood clotting time. As concerns pluronics, the data about their influence on blood coagulation are scarce. We were not able to find any data about effects of P123 on blood clotting. However, it was shown that homologous pluronics P105 and F68 (poloxamer 188) manifested weak anticoagulant activity, which was two orders of magnitude lower than that of heparin [35]. Moreover, pluronic F-68 interfered with platelet aggregation [36]. It is quite possible that the combination of two polymer anticoagulants may increase their activity. Indeed, in this work, the clotting time in the presence of Hep-P123 nanogel was prolonged compared to that of heparin. This fact may also be explained by a high heparin density in the Hep-P123 that led to enhanced interaction of the heparin with the proteases in the coagulation cascade. Hep-P123 nanogel was formed through a self-assembly process that is governed by the interactions of hydrophobic polymer domains in amphiphilic temperature-sensitive copolymers as the ambient temperature changed. These copolymers have been effectively utilized in drug delivery systems [7,15,20,37,38]. Regarding the Hep-P123 structure, the copolymer exists as a highly negatively-charged platform due to the abundance of anionic groups on the heparin chain. Such platforms have proven effective in the delivery of positive-charged bioactive molecules [7,20,39]. Ado and IW anticoagulants possessing free amino groups may be protonated to form positive-charged molecules, thus enhancing interaction with Hep-P123 copolymers (as illustrated in Figure 4), which then results in an increase of drug loading efficiency. Thus, the incorporation of the Ado or IW in the Hep-P123 nanogels may lead to protection of incapsulated molecules from degradation.

The structural and morphological characteristics of both free and loaded Hep-P123 showed the increase in nanoparticle size that suggested the encapsulation of anticoagulant. Indeed, the in vitro release studies indicated that Hep-P123 entrapped anticoagulant and retained them efficiently. The drug entrapment efficiency was fairly high and equal to 95.66 and 69.76 wt% for IW and Ado, respectively. The release of anticoagulant was not complete even after 96 hours. Encapsulation of both IW and Ado resulted in strong increase of their anticoagulant activity suggesting a good protection of anticoagulant from degradation. Encapsulated IW showed a stronger increase in activity than Ado. Higher increase in IW activity as compared to Ado may be explained by more effective protection from degradation at encapsulation in Hep-P123.

So the anticoagulants encapsulated in nanogel have been prepared and characterized. This encapsulation resulted in a strong increase in their anticoagulant activity. The results obtained indicate that Hep-P123 nanomaterial can be used for efficient protection and sustained delivery of drugs.

## 5. Conclusions

Hep-P123 nanogels were prepared and studied as carriers for anticoagulant delivery. The obtained results demonstrated that the heparin-based carriers possessed high Ado and IW loading efficiency. The in vivo studies showed that incorporation of the Ado or IW in the Hep-P123 nanogels resulted in substantially prolonged clotting and bleeding times. The Ado or IW-loaded Hep-P123 nanogels deserve further studies to clarify their anticoagulant mechanisms in an effort to pave the way for biomedical applications.

## Figures and Tables

**Table 1:** Cumulative anticoagulant release in in vitro experiments.

Cumulative Anticoagulant Release (%)				
Time (h)	IW	Ado	Hep-P123-IW	Hep-P123-Ado
1	35.17 ± 1.27	36.72 ± 5.54	6.48 ± 1.48	4.67 ± 2.40
3	52.08 ± 0.85	43.80 ± 4.73	12.87 ± 3.01	9.03 ± 2.03
5	59.20 ± 0.70	56.53 ± 6.84	18.52 ± 1.92	18.06 ± 3.61
7	85.86 ± 1.93	75.65 ± 10.10	22.95 ± 3.63	32.94 ± 4.52
9	87.13 ± 3.82	93.94 ± 10.14	28.44 ± 3.62	37.81 ± 5.03
12	93.84 ± 1.05	100.0 ± 9.10	33.11 ± 4.35	40.25 ± 4.04
24	100.0 ± 4.51	-	36.58 ± 7.26	45.87 ± 4.71
48	-	-	43.52 ± 2.64	47.59 ± 5.53
96	-	-	65.94 ± 5.28	57.74 ± 4.30
